# Editorial: Unsolved challenges in hepatitis B and hepatitis C: host-virus interaction, molecular pathogenesis and treatment strategies

**DOI:** 10.3389/fcimb.2025.1581433

**Published:** 2025-03-20

**Authors:** Peng Huang, Amei Zhang, Ming Yue

**Affiliations:** ^1^ Department of Epidemiology, Center for Global Health, School of Public Health, Nanjing Medical University, Nanjing, China; ^2^ Faculty of Life Science and Technology, Kunming University of Science and Technology, Yunnan, China; ^3^ Department of Infectious Diseases, The First Affiliated Hospital with Nanjing Medical University, Jiangsu Province Hospital, Nanjing, Jiangsu, China

**Keywords:** hepatitis B virus, hepatitis C virus, hepatitis D virus, prevention and control strategies, molecular mechanisms

Hepatitis B virus (HBV) and hepatitis C virus (HCV) infection remain the main causes of chronic liver disease worldwide. According to the latest WHO report, as of 2022, there are about 254 million HBV and 50 million HCV infections worldwide, with 1.3 million deaths each year, 83% caused by hepatitis B and 17% by hepatitis C. Despite the progress in prevention and control measures, the WHO’s goal of eliminating hepatitis by 2030 still faces severe challenges, which need to be coordinated in vaccination, treatment coverage and vaccine research and development.

The Research Topic: *Unsolved Challenges in Hepatitis B and Hepatitis C: Host-Virus Interaction, Molecular Pathogenesis and Treatment Strategies* of Journal: Frontiers in Cellular and Infection Microbiology focuses on hepatitis B, C, and D, aiming to investigate the interaction between these viruses and the host, elucidate pathogenic mechanisms at the molecular level, and provide insights into strategies for prevention and treatment.

Many countries have made continuous efforts in the prevention and control of hepatitis B. Hepatitis B infection is an important public health problem in China, and blood screening is crucial to ensure the safety of blood transfusion and reduce the risk of hepatitis B transmission. When Wei Hu et al. screened blood donors using transcription-mediated amplification, they found some non-discriminatory reactive results (i. e., reactive screening test but negative HBV-DNA discrimination test), which raised concerns about false positive donors and the impact of blood supply. In order to clarify the infection status of these non-discriminatory reactive blood donors, the Wei Hu et al. team carried out a series of supplementary tests based on the risk assessment and strategy optimization of hepatitis B infection by blood screening, and recorded multiple clinical variables of donors. Based on these data, the prediction model constructed using the progressive forward algorithm shows high discriminant power, good fit, university accuracy and validity. After comparing various screening strategies through cost-benefit analysis, it was found that the strategy of “two repeated identification tests + model prediction + reentry test” was the most cost-effective, which could not only effectively identify the infection status of non-discriminatory reactive blood donors, but also guarantee the blood supply and maximize the benefit cost ratio of.

Since China included the hepatitis B vaccine in its national immunization program in 2002, the three-dose vaccination rate among newborns has been high, and the results have been remarkable. Shanghai’s Huangpu District, as a key area for hepatitis B prevention and control in China, has a significant impact on understanding the effectiveness of hepatitis B prevention and control in urban areas of China. Wang et al. conducted a cross-sectional survey among people aged 1-69 in Huangpu District, and the results showed that the HBsAg positivity rate among children aged 1-14 had dropped to 0%, achieving the relevant goals of the WHO Western Pacific region ahead of schedule. However, there are shortcomings in the timeliness of vaccination. For example, among children aged 1-14 in Huangpu District, the HBcAb prevalence was higher in those who received the second dose of the vaccine more than 30 days late than in those who received it on time. In addition, the HBsAg positivity rate among people aged 15-69 was still as high as 4.21%, and the HBcAb positivity rate was relatively high. Multivariate analysis showed that age (50-69 years old) and HBsAb positivity were risk factors for HBcAb positivity, while higher education was a protective factor. Moreover, among HBsAg - positive individuals, only 16.67% received antiviral treatment, and 33.33% had the risk of household transmission, highlighting the importance of managing chronic infections.

In Spain, the awareness of hepatitis B within the Chinese immigrant community poses challenges for the management of chronic HBV infection. A study conducted by Pocurull et al. in Barcelona, Spain, explored the impact of language barriers and cultural differences on hepatitis B knowledge among the local Chinese community. The findings revealed that 80% of Chinese patients and 44% of their family members faced language barriers. Although Chinese patients demonstrated higher awareness of vertical HBV transmission compared to non-Chinese counterparts (94% vs. 68%), the HBV vaccination rate among Chinese family members (78%) was slightly lower than that of non-Chinese families (86%), particularly among those born in China (59% vaccination rate). This highlights the need to reinforce HBV vaccination promotion efforts targeting Chinese families, especially those born outside Spain.

Africa faces more hepatitis B prevention and control challenges.  Torimiro et al. reported that in some countries, the cold chain transportation of vaccines is difficult to guarantee, resulting in the first dose vaccination rate of newborns being less than 60%, and the positive rate of HBsAg in children is still as high as 8-15%. At the same time, only 30% of HBsAg positive pregnant women receive prenatal screening, the utilization rate of hepatitis B immunoglobulin is less than 10%, and the problem of mother-to-child transmission is prominent, which is an important contributing factor to the transmission of hepatitis B. Moreover, the early diagnosis system of liver cancer in this region is missing, 80% of liver cancer cases are advanced when diagnosed, and the 5-year survival rate is less than 10%.

The above studies in different countries present the multifaceted nature of global hepatitis B prevention and control. China’s vaccination strategies are effective, but they still need to be strengthened in adult catch-up vaccination and management of chronic infections; Africa is limited by economic and medical resources, with a significant gap in improving vaccination coverage and early diagnosis and treatment of hepatitis B; and the Spain study highlights the impact of language and cultural barriers on hepatitis B control. Collectively, these findings underscore that effective global hepatitis B control requires a multifaceted approach integrating regional variations in resource availability, sociocultural contexts, and genetic epidemiology to formulate context-specific intervention frameworks.

Pisaturo et al. presents the latest research advancements in treatment strategies for chronic hepatitis D. The hepatitis D virus (HDV) is a defective RNA virus that relies on the hepatitis B virus surface antigen (HBsAg) for assembly and enters hepatocytes by binding to the sodium taurocholate co-transporting polypeptide (NTCP), a shared cellular receptor for both HDV and HBV. Patients co-infected or superinfected with HDV and HBV experience worse clinical outcomes, including higher rates of complications such as cirrhosis, hepatocellular carcinoma, and hepatic decompensation. Global epidemiological data on HDV infection vary due to limited surveillance, with an estimated HDV prevalence of approximately 0.80% to 0.98% in the general population and 13.02% to 14.57% among HBsAg-positive individuals [1]. Traditional treatment strategy for hepatitis D is pegylated interferon (PegIFN)-based therapy, which has limited efficacy, high relapse rates, and significant side effects. Exciting innovations in HDV therapeutics have emerged in recent years. Novel agents like bulevirtide (BLV), a peptide-based entry inhibitor that blocks the binding of HBsAg-enveloped particles to NTCP and prevents HDV entry into hepatocytes, have arisen. BLV has demonstrated promising results in clinical trials and has received conditional marketing authorization in Europe. It can be administered either in combination with PegIFN or as monotherapy, showing significant improvements in virological and biochemical responses. Real-world studies confirm the effectiveness of BLV, highlighting its ability to reduce HDV RNA levels, improve liver function, and maintain a favorable safety profile. Overall, HDV-related chronic hepatitis is increasingly becoming a potentially curable disease, with novel therapies offering hope for improved outcomes. Future research should focus on developing more effective and safer HDV treatment regimens while strengthening epidemiological surveillance to better cope with this challenging disease.

The study by Bunz et. al. revealed a new role of CD81 in HCV infection: it regulates host cell survival and viral persistence by inhibiting the NF-κB signaling pathway, providing a breakthrough perspective for understanding the pathogenic mechanisms of HCV. CD81 is one of the main entry receptors for HCV and belongs to the tetraspanin family, playing an important role in cell signaling. The study found that HCV replication can specifically downregulate the expression of CD81 on the surface of liver cells, and this phenomenon is driven by the viral non - structural protein NS5A at the transcriptional level. The absence of CD81 not only accelerates HCV protein expression but also enhances TBK1 - dependent survival signals to resist apoptotic pressure. Further mechanistic studies have shown that HCV downregulates CD81 to relieve its inhibitory effect on NF-κB subunits p50/p65, promoting the proliferative advantage of infected cells. This process may be closely related to hepatocellular carcinogenesis in the chronic infection microenvironment. The research updates the biological functions of CD81 and reveals the “bidirectional regulation” strategy of HCV - - reducing surface receptors to limit superinfection while activating NF-κB - dependent pro-survival pathways to maintain viral persistence. This finding provides a theoretical basis for the development of combination therapies targeting the CD81 - NF-κB axis (such as small molecules mimicking CD81 functions or gene editing interventions). In the future, it is necessary to verify its universality in primary hepatocyte models and clinical cohorts and explore how CD81 precisely regulates upstream kinases (such as IKKβ/TBK1) to deepen the molecular mechanism. This study fills the gap in the understanding of the chronicization mechanism of HCV and lays a scientific foundation for the development of treatment strategies with both antiviral and anti -tumor effects.

In conclusion, the prevention and control of viral hepatitis remains a daunting task globally, facing numerous challenges, but it is also continuously making new progress. The global prevention and control of viral hepatitis require interdisciplinary and cross - border cooperation, integrating resources from all parties, and advancing comprehensively from basic research to clinical application, and from vaccine research and development, vaccination to chronic disease management. Only in this way can we gradually achieve the global public health goal of eliminating viral hepatitis and make greater contributions to the human health cause.

